# High throughput single cell counting in droplet-based microfluidics

**DOI:** 10.1038/s41598-017-01454-4

**Published:** 2017-05-02

**Authors:** Heng Lu, Ouriel Caen, Jeremy Vrignon, Eleonora Zonta, Zakaria El Harrak, Philippe Nizard, Jean-Christophe Baret, Valérie Taly

**Affiliations:** 1 INSERM UMR-S1147, CNRS SNC5014, Paris Descartes University, Equipe labellisée Ligue Nationale contre le cancer, Paris, France; 2 CNRS, Univ. Bordeaux, CRPP, UPR 8641, 115 Avenue Schweitzer, 33600 Pessac, France

**Keywords:** Lab-on-a-chip, Engineering

## Abstract

Droplet-based microfluidics is extensively and increasingly used for high-throughput single-cell studies. However, the accuracy of the cell counting method directly impacts the robustness of such studies. We describe here a simple and precise method to accurately count a large number of adherent and non-adherent human cells as well as bacteria. Our microfluidic hemocytometer provides statistically relevant data on large populations of cells at a high-throughput, used to characterize cell encapsulation and cell viability during incubation in droplets.

## Introduction

Droplet-based microfluidics has emerged as a powerful tool in a large spectrum of applications ranging from fundamental biological research^1–3^ to clinical research^4–6^. An important feature of this technology is to access single-cell phenotypic informations in a high-throughput fashion for applications such as single-cell based enzymatic assays^7–12^, drug susceptibility assessment^13–15^ and single-cell DNA or RNA sequencing^1–3^. In droplet-based microfluidics single-cell studies, reliable methods to measure the cell occupancy are rarely described (Supplementary Table S1). Two main methods are traditionally used to detect cells in droplets: image-based analysis and laser induced fluorescence (LIF). Image-based analysis provides detailed information related to the shape as well as the cell physiological condition when combined with fluorescent assays. This high-content measurement is however often incompatible with high-throughput analysis. Indeed the implementation of fast and automatized image analysis algorithms to detect cells is challenging and most studies rely on manual counting of small population of cells^2, 3, 16–18^. In addition, fluorescence imaging is mainly achievable for limited size arrays of cells or droplets^19^ as it requires long exposure times and immobilization of droplets on-chip. In contrast, LIF is usable for high-throughput analysis as each droplet and encapsulated cell is continuously scanned by a laser and fluorescence signals are measured by a photomultiplier tube: the signal readout is reduced to a single parameter (low-content) and therefore lacks information related to shape but the throughput is then drastically increased to tens of thousands of measurements per second^20^. Cell encapsulation follows a Poisson distribution, as expected for randomly dispersed objects^7, 13, 17, 18^. Consequently if the cell concentration is ≤0.1 cell per droplet, single-cell droplets will account to at least 95% of the non-empty droplets^2, 3^. In this case the counting process is relatively straightforward. However, increasing the cell occupancy is of interest to increase the screening throughputs^13, 21^ or to study different cell lines within a same assay^22^. A data analysis method allowing to detect cells within a droplet at high density becomes essential. Only few approaches have been described regarding this issue. One solution is to set up two thresholds to detect the droplet and its cell occupancy respectively^7, 13, 15^ or even three thresholds^22^, one for the droplets, and two to indicate respectively the rising edge and falling edge of cell signals. Moreover, as all these methods are solely based on thresholding, their degree of accuracy is limited. Their use is not suited for instance to detect cells in close proximity or cell aggregates. This is a strong limitation as this situation often occurs for cell lines growing as aggregates or during mitosis.

Here we describe a LIF-based procedure to accurately count cells in droplets by overcoming counting errors caused by signal noise, cells in close proximity and cell clumping. The data acquisition and analysis were optimized to analyse large amounts of data in short time (~20 min per dataset) using readily implementable tools. As a proof of principle, we used this method to characterize the encapsulation of adherent human cells, non-adherent human cells (~10 µm diameter) and bacteria (~1 µm diameter). We further describe a protocol for a precise assessment of mammalian cell viability and proliferation. Altogether, our procedure could improve the robustness of droplet-based microfluidics single-cell studies.

## Results and Discussion

**Figure 1:**
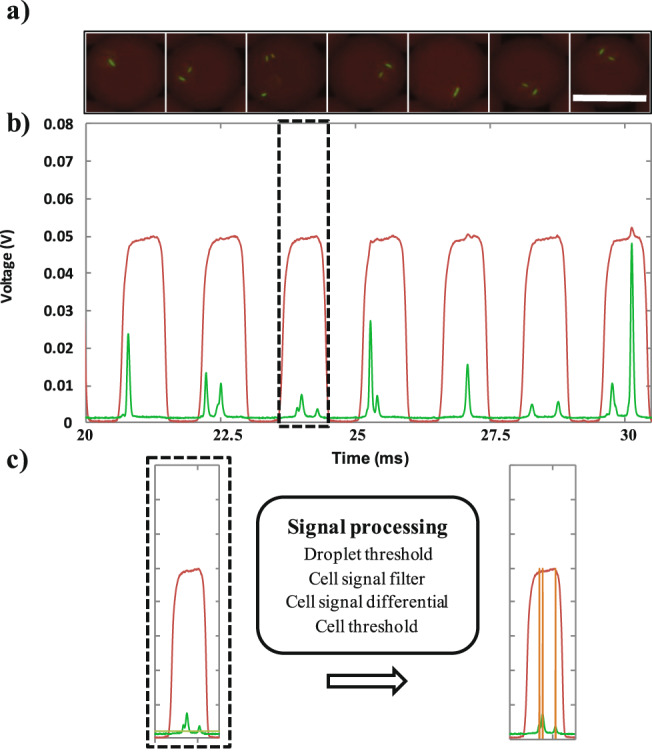
Schematic representation of the cell counting procedure. eGFP-transformed *E. coli* cells were encapsulated in droplets. (**a**) Fluorescent images of red coded droplets encapsulating *E. coli* cells at λ = 2 (mean cell per droplet ratio). Scale bar: 30 µm. Corresponding time sequence of red and green fluorescence signals is shown in (**b**). Dashed black rectangle encloses a signal sample corresponding to a droplet chosen as an example to illustrate the signal processing analysis. (**c**) The signal processing method is schematized in the black box. Briefly, each droplet is identified by applying a droplet threshold on the red fluorescence channel. The green fluorescence channel was then filtered within each droplet, and a first order differential is applied to identify the local maximal values. A cell threshold (grey line) is eventually applied to identify cells. The number of cells per droplet is then enumerated as signal peaks (orange) within the interval of each droplet. An exhaustive description of the process can be found in Supplementary Fig. S1.

We first tested the validity of our counting method by using it to analyze several time series of droplets and cells signals. Peaks within encoded droplets could be properly identified as cells using the developed data analysis procedure (Fig. 1). A more detailed description of the analysis process can be found in Supplementary Fig. S1. In contrast to the traditional thresholding-based methods, cells in close proximity or clumped together could be discriminated and counted individually (Fig. 1c). On average, the droplet detection rate was ~570 Hz. At a mean occupational rate of 1 cell per drop, for instance, the detection time of 100,000 cells is thus ~3 minutes. Moreover it can be expected that droplet detection rate could be further increased to several kHz yet it would require to increase the acquisition sampling frequency. The total experimental time including cell preparation, microfluidic setup preparation, experiment and data analysis is ~1.5 hour. A similar experimental time allowed us to count only ~100 cells using epifluorescence imaging.

### Counting of *E. coli* cells

**Figure 2:**
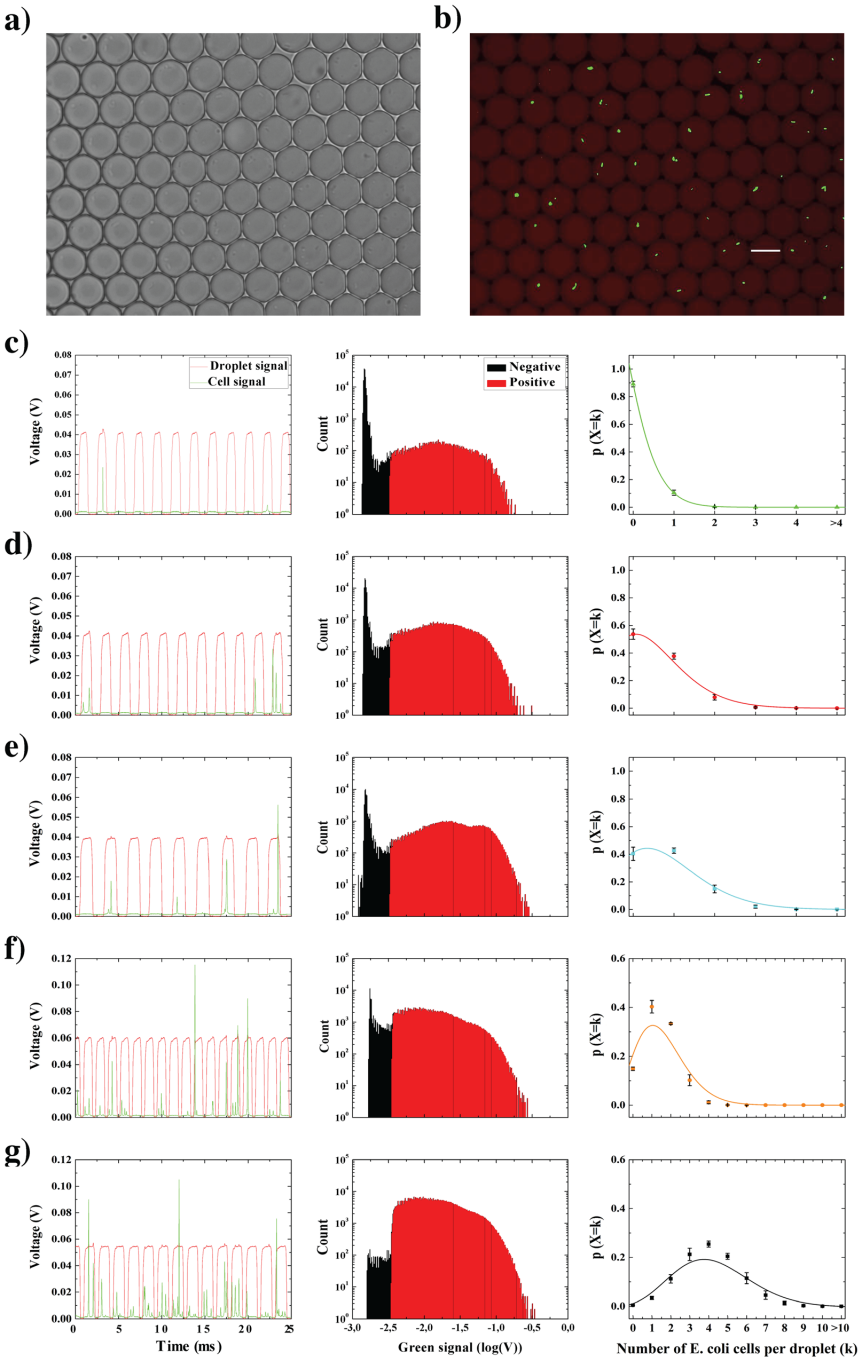
Counting of *E. coli* cells. Bright field image (**a**) and fluorescence image (**b**) of eGFP transformed *E. coli* cells encapsulated in droplets. The droplets were labeled by adding the soluble dye Sulforhodamine-B in the aqueous phase. Scale bar: 30 µm. (**c**–**g**) From left to right: time sequences of red and green fluorescence signals, histograms of the green fluorescence signal depicting negative and positive cell count events, and cell distribution in droplets (mean ± s.d for n = 3 (**c**–**e**) and n = 2 (**f**,**g**)); Poisson fit is plotted as a straight line). (**c**) Cell density was adjusted to 2 × 10^6^ cells/mL such that expected theoretical cell to droplet ratio (λ_theo_) is λ_theo_ = 0.1 (given that droplet’s volume is 14 pL). On average 19,698 ± 3,911 cells were counted out of 175,254 ± 36,027 droplets resulting an experimental cell to droplet ratio (λ_exp_) λ_exp_ = 0.11 ± 0.02. Cell distribution fitted λ_fit_ = 0.1 ± (1 × 10^−3^) with R^2^ = 0.99 (R: coefficient of determination). (**d**) Cell density was adjusted to 1.05 × 10^7^ cells/mL such that λ_theo_ = 0.5 is expected. On average 108,486 ± 15,084 cells were counted out of 195,886 ± 57,982 droplets resulting in λ_exp_ = 0.55 ± 0.08. Cell distribution fitted λ_fit_ = 0.62 ± (4 × 10^−2^) with R^2^ = 0.99. (**e**) Cell density was adjusted to 2.1 × 10^7^ cells/mL such that λ_theo_ = 1 is expected. On average 180,206 ± 25,995 cells were counted out of 228,015 ± 98,897 droplets resulting in λ_exp_ = 0.79 ± 0.11. Cell distribution fitted λ_fit_ = 0.9 ± 0.07 with R^2^ = 0.97. (**f**) Cell density was adjusted to 1.05 × 10^8^ cells/mL such that λ_theo_ = 2 is expected. On average 286,374 ± 25,382 cells were counted out of 200,850 ± 14,296 droplets resulting in λ_exp_ = 1.43 ± 0.13. Cell distribution fitted λ_fit_ = 1.57 ± 0.15 with R^2^ = 0.91. (**g**) Cell density was adjusted to 4.2 × 10^8^ cells/mL such that λ_theo_ = 5 is expected. On average 731,518 ± 102,214 cells were counted out of 179,058 ± 16,523 droplets resulting in λ_exp_ = 4.08 ± 0.57. Cell distribution fitted λ_fit_ = 4.2 ± 0.22 with R^2^ = 0.9.

Plasmid and cell culture protocol are described in Supplementary Information. Before encapsulation in droplets, the cell densities were adjusted to 2 × 10^6^, 1.05 × 10^7^, 2.1 × 10^7^, 1.05 × 10^8^ and 4.2 × 10^8^ cells/mL, respectively. The cell distribution in droplets fitted a Poisson distribution with R^2^ = 0.98 for the three first cell densities (Fig. 2c–e). However, for the two highest cell densities, the Poisson fit correlations were slightly lower: R^2^ = 0.91 and R^2^ = 0.9, respectively (Fig. 2f and g). These two densities correspond, in 14 pL droplets, to an expected mean cell per droplet ratio (λ) of 2 and 5 respectively. For the latter densities the probability of droplets to contain more than 2 and 5 cells respectively is lower than expected by the Poisson distribution. Conversely, the probability of droplets to contain less than 2 and 5 cells respectively is higher than expected. This shift clearly indicates a lack of precision regarding the counting of cells in highly occupied droplets (λ > 1). Such slight discrepancies can be explained by variations in fluorescence signal amplitude due to variations of the cell position within the droplet. The counting accuracy is more sensitive to such variations at high densities in which the occurrence of overlapping cell peaks signal is more likely. Our procedure however allows to limit the latter effects on counting accuracy by recovering the integrality of the fluorescence signals. Thus, a careful analysis and treatment of data allows an optimal filtering of noise (see data analysis section and Fig. S1). Moreover, we show that a potentially major source of errors caused by overlapping cells and cells in close proximity is overcome by our method. We performed supplementary analyses to directly compare a traditional peak detection approach, relying on a simple cell threshold, with the differential-based approach presented in our work (Figs S2 and S3). We considered the highest cell occupancy rate per droplet (λ = 5) scenario as it is likely to observe overlapping cells and cells in close proximity in this configuration. Within Fig. S2 we show the detailed analysis of a series of droplets and cells fluorescence signals. The traditional peak detection approach shows clear discrepancy with expected cell count per droplet. Contrariwise, the differential-based cell signal detection used with our approach is fully consistent with expected counts. Moreover, Fig. S3 describes cell distributions on larger datasets (more than half a million of cells, replicated experiments). It can clearly be seen that the analysis performed with the differential-based approach allows to plot a distribution which is in closer agreement with theory than the classic approach. It is also interesting to note that optical optimizations can allow to further minimize fluorescence variations due to cell positioning in the droplet. In particular, the use of a laser line larger than the flow channel width allows, contrarily to a traditional laser spot, to fully scan the droplet (see Methods section and Figs S8 and S9) and hence help in limiting error counts. It can be assumed that further improved quantifications could be obtained by increasing the signal sampling frequency. At a given droplet injection frequency, the use of larger droplets would for instance allow the recording of a larger number of photons. Such an approach could enable to reach an improved resolution of droplets and cells signals. In particular, this could allow a better discrimination of overlapping cell peaks signals in high occupancy rate situations. In the same logic, another approach would consist in increasing recovered fluorescence signals resolution by using electronic components with larger frequency bandwidth. Such a solution however implies financial costs which should be taken in consideration.

### Counting of human cells

**Figure 3:**
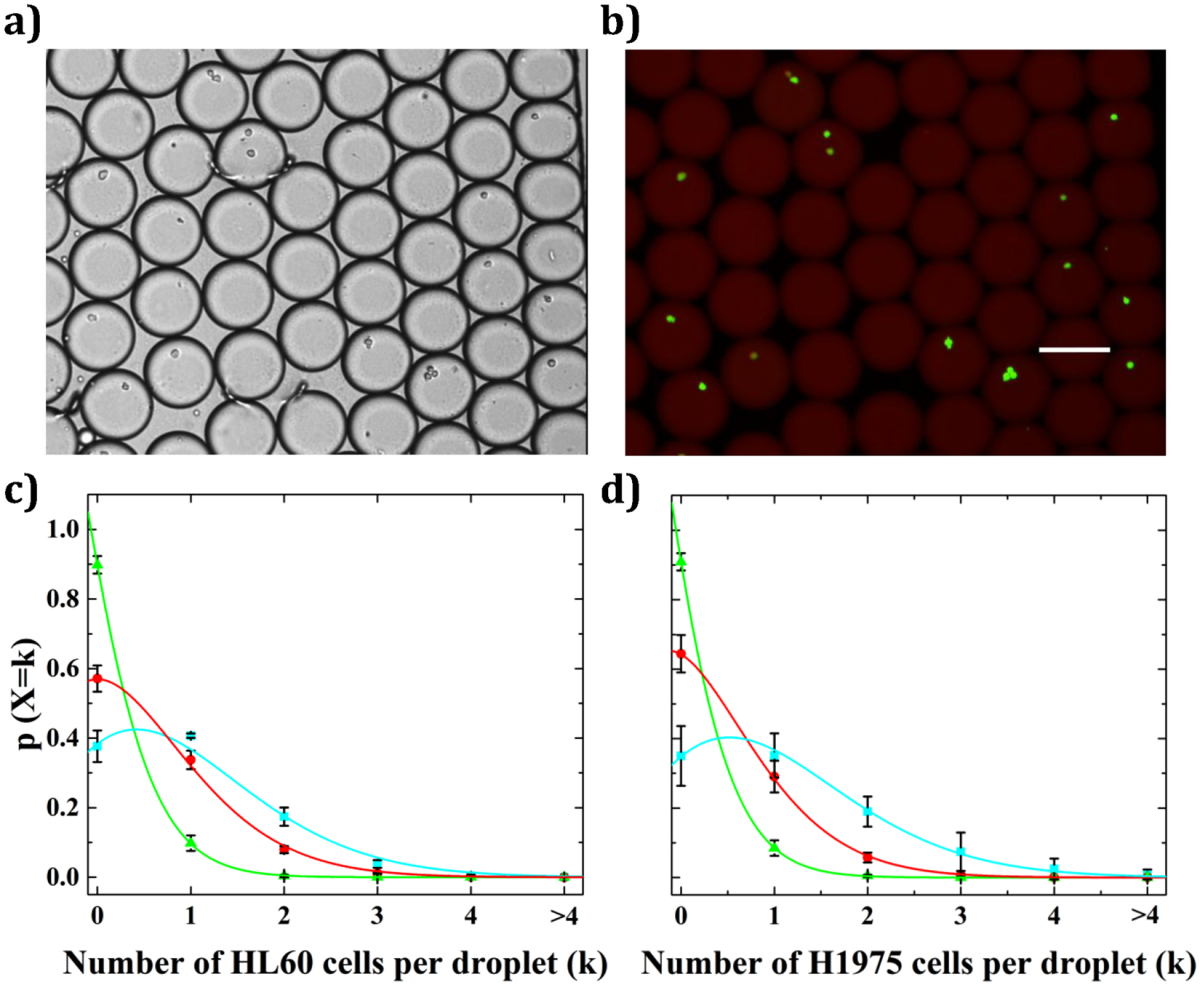
Counting of human cells. Bright field image **(a)** and fluorescence image **(b)** of HL60 cells encapsulated in droplets. Droplets were labeled by adding the soluble dye Sulforhodamine-B in the aqueous phase. Scale bar: 100 µm. **(c)** Distribution of HL60 cells in droplets (mean ± s.d for n = 3; Poisson fit is plotted as a straight line). Green triangles. Cell density was adjusted to 2 × 10^5^ cells/mL such that expected theoretical cell to droplet ratio (λ_theo_) is λ_theo_ = 0.1 (given that droplet’s volume is 500 pL). Cell distribution fitted λ_fit_ = 0.1 ± (7.4 × 10^−4^) with R^2^ = 0.99. Red circles. Cell density was adjusted to 10^6^ cells/mL such that λ_theo_ = 0.5. Cell distribution fitted λ_fit_ = 0.56 ± 0.01 with R^2^ = 0.99. Blue squares. Cell density was adjusted to 2 × 10^6^ cells/mL such that λ_theo_ = 1. Cell distribution fitted λ_fit_ = 0.96 ± 0.01 with R^2^ = 0.98. **(d)** Distribution of H1975 cells in droplets (mean ± s.d for n = 3; Poisson fit is plotted as a straight line). Green triangles. Cell density was adjusted to 2 × 10^5^ cells/mL such that λ_theo_ = 0.1. Cell distribution fitted λ_fit_ = 0.1 ± 0.006 with R^2^ = 0.99. Red circles. Cell density was adjusted to 10^6^ cells/mL such that λ_theo_ = 0.5. Cell distribution fitted λ_fit_ = 0.44 ± 0.006 with R^2^ = 0.99. Blue squares. Cell density was adjusted to 2 × 10^6^ cells/mL such that λ_theo_ = 1. Cell distribution fitted λ_fit_ = 1 ± 0.02 with R^2^ = 0.99.

Cell culture protocol are described in Supplementary Information. HL60 (human promyelocytic leukemia cells, non-adherent) and H1975 (non-small cell lung cancer cells, adherent) densities were adjusted to 2 × 10^5^, 10^6^, and 2 × 10^6^ cells/mL, respectively. The cell encapsulation in droplet at the highest density is shown in Supplementary video S1. The experiments were not performed for higher cell concentrations since the cell growth plateau density was then reached. The cell distribution in droplets followed Poisson statistics with a high coefficient of determination R^2^ = 0.98 (Fig. 3c and d). More detailed statistics can be found for HL60 and H1975 cell lines in Supplementary Figs S5 and S6, respectively. All experiments were independently reproduced three times and at least 100,000 droplets were analyzed per experiment. Such a high statistical significance confirms the robustness and accuracy of our method. Some discrepancy is however noticeable between expected cell to droplet ratio values and those measured experimentally. Indeed from the lowest to the highest cell densities, a mean percentage error of 0%, 10% and 8.5% is respectively obtained. We assume such variations are due to experimental inaccuracy as variations in cell densities from the manual cell counting procedures are expected, especially at high densities.

### Cell viability and proliferation in droplets

**Figure 4:**
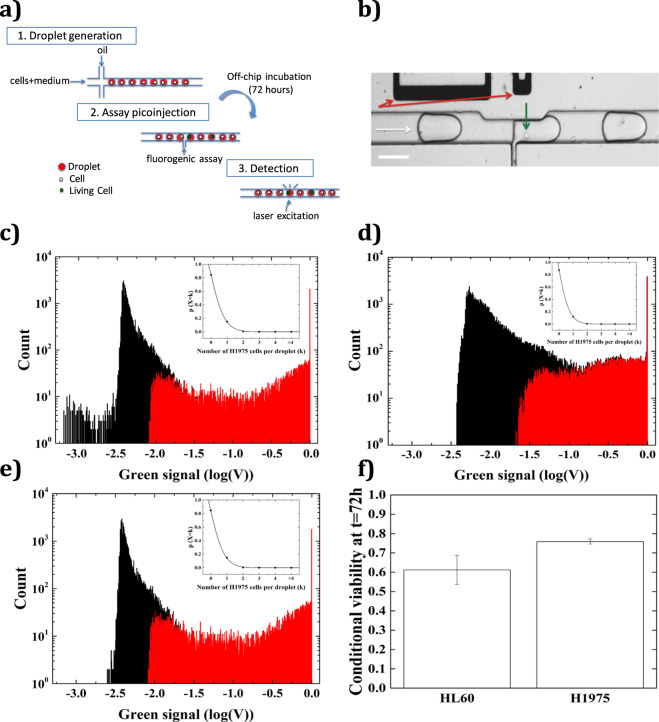
Cell viability. (**a**) Schematic representation of the microfluidic workflow used for the characterization of H1975 and HL60 cells viability in droplets. Cells were encapsulated in droplets with medium and incubated for 72 hours. A fluorogenic assay (Calcein-AM) was then injected in each droplet allowing the fluorescent detection of the droplets containing living cells. (**b**) Image of the electro-microfluidic injection system. The white arrow indicates the droplet flow direction. The green arrow indicates the presence of a cell. When the droplet passes by the injection arm, it is locally destabilized by applying an electric field. The field ruptures the film separating the droplet and reagent, allowing the reagent to be injected. Scale bar: 100 µm. (**c**–**e**) Green fluorescence histograms depicting negative (black) and positive (red) cell count events. (**c**) Signal recorded at cell encapsulation with the fluorogenic assay added in bulk. 9,150 cells were counted out of 56,567 droplets resulting in λ = 0.16. Inset shows cell distribution with Poisson fit as a straight line: λ_fit_ = 0.17 ± 0.004 with R^2^ = 0.99. (**d**) Signal recorded 72 hours after cell encapsulation. 13,865 cells were counted out of 105,781 droplets resulting in λ = 0.13. Inset: λ_fit_ = 0.14 ± 0.002 with R^2^ = 0.99. (**e**) Signal recorded at encapsulation with the fluorogenic assay added drop-by-drop using the electro-microfluidic device. 8,127 cells were counted out of 51,054 droplets resulting in λ = 0.16. Inset: λ_fit_ = 0.17 ± 0.004 with R^2^ = 0.99. (**f**) HL60 and H1975 conditional viability following 72 hours of incubation in droplets. HL60: λ_t=72h_/λ_t=0h_ = 0.61 ± 0.07 (mean ± s.d, n = 2). H1975: λ_t=72h_/λ_t=0h_ = 0.77 ± 0.04 (mean ± s.d, n = 3).

We designed a microfluidic workflow to investigate the cell viability in droplets following 72 hours of incubation (Fig. 4a). This incubation time was chosen since both HL60 and H1975 cells undergo at least a full division cycle within this time frame, as observed in bulk. To maximize cell survival prior to encapsulation the cell dilution was held in a dry bath at 37 °C. For each experiment one portion of the cell suspension was stained by Calcein-AM and emulsified to assess the cell-to-droplet ratio at encapsulation (Fig. 4c). The remaining portion of cells were encapsulated and incubated off-chip at 37 °C and 5% CO2. After 72 h of incubation, the droplets were injected one by one with Calcein-AM by the use of an optimized version of a previously described electro-microfluidic technology^23^ (Fig. 4b). Such drop-by-drop injection of the viability assay can be seen in Supplementary video S2. We controlled the robustness of this droplet injection operation by verifying that the measured cell distribution matched with the one obtained when the fluorogenic assay was added in bulk (Fig. 4e). This also indicates that the electric field has little effect on the cells which can be explained by the fact that cells flow in the high field region only for a time shorter than a millisecond. Droplets were then reinjected into a detection module for fluorescence measurements (Fig. 4d). The cell-to-droplet ratio after 72 h was then evaluated and normalized to the ratio obtained at encapsulation. H1975 and HL60 cells exhibited a mean normalized ratio of 0.77 ± 0.04 and 0.61 ± 0.07 respectively (Fig. 4f). Previous studies have analyzed the survival rates in droplets of Jurkat cells (human T lymphocyte cells), Human Embryonic Kidney 293 T cells (HEK293T)^18^ and human monocytic U937 cells^13^. In those studies, the measured survival rates following 72 h of incubation in droplets were close to or higher than 80%. However significant variations (~10%) could be found depending on the studied cell lines (Jurkat vs HEK293T). The measured survival rates in our study were hence reasonably consistent with these previous results regarding H1975 cells but lower regarding HL60 cells. During encapsulation, cells are flowing in narrow channels in which the shear stress might impact on the cell membrane. The effect of the shear rate on the membrane of the cell is probably cell dependent and could explain the differences between the cell lines. However, our analysis method demonstrated a higher statistical significance compared to these previously described works. Indeed the studies mentioned above counted several hundreds of cells, a sample size a hundred fold smaller than in our study (<n> = 27,296 analyzed cells per experiment). It can also be highlighted that our procedure allows to access information regarding both cell viability and cell proliferation from a same experiment. Proliferation can indeed be inferred from data analysis as cell distribution in droplets can be precisely extracted. In our case, no significant increase was observed following incubation regarding occupational rates of 2 or more cells per droplet. Therefore no proliferation seems to occur in droplet for both the two cell lines. Such a result is consistent with formerly published data obtained with LIF (see ref. 13). This limited proliferation could be explained by the fact that the effective density of 1 cell in a 500 pL droplet is equivalent to ~2 million cells/mL at which cell growth is limited.

## Conclusion

We developed a LIF-based procedure to efficiently count cells in droplets, even in the presence of multiple encapsulations. We demonstrate the accurate counting of various cell types within droplets. We first verified that the distribution of bacteria and human cells within droplets follows expected Poisson statistics with a high consistency. We further assessed human cell viability in droplets using this method. Compared to similar viability studies, our measurements are highly quantitative as the number of counted cells was a hundred fold higher than previously described. In the long-run the data analysis algorithm could be embedded in the LabVIEW FPGA software for real-time analysis and droplet sorting^24^. We believe that our procedure improves the quantitativity of droplet-based single-cell studies and provides a clear protocol to assess the survival rate of cells in droplets, a pre-requisite for most cell-based assays. The presented method has clear advantages over traditional cell counting methods such as imaging or flow cytometry. Indeed imaging is only usable with adherent cells and flow cytometry does not allow the screening automation offered through droplet fluorescence coding.

## Methods

### Microfluidic experiments

HL60 (human promyelocytic leukemia cells, DMSZ) and H1975 (non-small cell lung cancer cells, ATCC) were encapsulated into ~500 pL droplets. Droplets were produced by flow focusing the aqueous phase with a fluorinated oil phase (HFE7500, 3 M) containing 2% (w/w) EA-surfactant (RainDance Technologies), a biocompatible PEG-PFPE amphiphilic blockcopolymer^25^. Sulforhodamine-B (Sigma-Aldrich) was added to the aqueous phase at 10 µM to fluorescently labeled droplets. The two phases were contained in 15 mL Falcons (Greiner bio-one) connected to a 15 mL Flowell (Fluigent) and driven by an MFCS pressure controler (Fluigent). In order to prevent cell sedimentation during encapsulation, the cell-containing Falcon was set on a vortex at 800 rpm. The two phases were injected into the microfluidic chips through Polyetheretherketone (PEEK) tubings (CIL Upchurch) and the falcon used for droplets collection was connected with a PolyTetraFluoroEthylene (PTFE) tubing (Fisher Scientific). The MFCS pressure was set at 220 mbar for the oil phase and 200 mbar for the cell suspension phase for *E. Coli* cells. For human cells the oil phase was pressurized at 180 mbar and the cell suspension phase at 150 mbar. Injection of individual droplets by Calcein-AM (Life technologies) at 10 µM was performed to stain living cells. This operation was allowed by the use of a previously described electro-microfluidic technology^23^. The MFCS pressure was set at 250 mbar for the aqueous and oil phases and 150 mbar for the Calcein-AM suspension phase. A 30 kHz sinusoidal voltage was generated using a signal generator (33521 A, Agilent) and amplified to 500 Vpp (623B, Trek) to be applied to the electrodes connected to the chip. *E. coli* cells were eGFP-transformed to allow fluorescence detection. The bacteria suspension was contained in a 2 mL tube (Fisher Scientific). The MFCS pressure was set at 630 mbar for oil phase and 560 mbar for the bacteria suspension phase. The droplets and cell fluorescence were simultaneously measured on chip thanks to a laser line optical set-up^21^ (see Figs S8 and S9) allowing the excitation of cells independently of their position in the droplets. When droplets were previously incubated the latter fluorescence read-out was performed within a detection module in which droplets were reinjected. The MFCS pressure was then set at 200 mbar for droplets and 220 mbar for the oil phase. Details regarding the microfluidic chips preparation can be found in the Microfluidic chip fabrication section below. The designs and dimensions of the devices are shown in Supplementary Fig. S7.

### Optical detection

The optical set-up is described in Supplementary Fig. S8. Objectives with 10× and 40× magnifications were used for the detection of mammalian cells and bacteria, respectively. A bright field image of the laser line excitation area can be found in Fig. S9.

### Data acquisition

The photomultiplier tubes (PMT) signals are recorded and converted to 8 bits through the Analog to Digital Converter of the LabVIEW FPGA module. The sampling frequency is adjusted on needs up to 200 kHz. At each acquisition step, both 8 bit PMT value are joined to form a 64 bit word queued in a LabVIEW direct memory access (DMA) first in, first out (FIFO). DMA FIFO enables high bandwidth transfer of data from the Field-programmable gate array (FPGA) to the host computer. Up to four 8 bit signals can be joined in these 64 bit words, so PMT acquisition can be transferred together with other available internal signal, depending on the user need (such as internal machine state, camera synchronization signal, and/or high voltage signal trigger). This usage of a monolithic 64 bits proved to increase robustness against data and/or synchronization loss in comparison to either serial 8 bit entrelacement or usage of parallel DMA FIFO. On the host computer FIFO elements are dequeued in the main LabVIEW vi. Each 64 bits word is decoded to the four original 8 bits values. These data are then directly streamed to the disk in a Waveform Audio FileFormat (WAV) 8 bit PCM. At this step, care is taken to dequeue data from the FIFO in a timely manner, in order to avoid any data loss. This involves the proper design of the execution structure of the overall LabVIEW vi to prioritize the host computer resources. The use of WAV 8 bit PCM as saving format has several advantages among which: (i) the possibility to record 4 audio tracks simultaneously while keeping synchronization. (ii) the uncompressed nature of the file which implies a lighter load on the central processing unit during recording and no data loss. (iii) the ability to include metadata in the header, such as the sampling frequency (iv) the availability of OpenSource visualization tools (Audacity) and interface library in several analysis language (C, Scilab, MATLAB). 500 MB of data in wav format (corresponding to 10 min) was recorded for each experiment. Audacity was used to convert the signal into 16 bits format and a home-made MATLAB script was further used to count the cells in each droplet.

### Data analysis

We developed a matlab script to count the number of cells in each droplet. It is composed of three steps. Firstly, the fluorescent signals of droplets (red channel) and cells (green channel) in the time course are filtered and illustrated in a 1D plot (Fig. 1c). This plot allows to define two thresholds, one for the detection of droplets and another for the detection of cells. The threshold of droplet should be higher than the noise level and lower than the droplets fluorescent level. Second, the droplet-passing time points are selected by applying the threshold of droplets (Supplementary Fig. S1a and b). As a last step, the signals of cells in each droplet are located within the droplet occurence dataset (Supplementary Fig. S1c). Within these sequences, the cell signal (Scell) is filtered by a triangular convolution window of length n with the kernel (1, 2, 3, … n …, 3, 2, 1) (Supplementary Fig. S1a,d). The length n of the kernel should be large enough to filter the noise, but at the same time not too large to avoid deteriorating the cell signal peaks. This convolution operation behaves like a low-pass filter to discard false positive peaks caused by noise within cell signal. In order to detect all the cell signal peaks within a droplet time series, a simple first-order derivative is applied to calculate the local maximum value (Supplementary Fig. S1e):

$${{\boldsymbol{S}}}_{{\boldsymbol{cell}}}^{{\boldsymbol{derivative}}}({\boldsymbol{i}})={{\boldsymbol{S}}}_{{\boldsymbol{cell}}}^{{\boldsymbol{filtered}}}({\boldsymbol{i}})-{{\boldsymbol{S}}}_{{\boldsymbol{cell}}}^{{\boldsymbol{filtered}}}({\boldsymbol{i}}-1)$$

These values are identified by the condition that the precedent derivative point is positive and successive point is negative. For elimination of the noise point, a minimum threshold for detecting these cells is applied (Supplementary Fig. S1f). The number of cells in every sequence can be calculated (Supplementary Fig. S1g). The complete MATLAB script is shown in Supplementary Fig. S4.

### Cell culture

HL60 cells were cultured in RPMI-1640 medium (LifeTechnologies) supplemented with 10% of heat-inactivated Fetal Calf Serum (FCS) (lot n°S11060S181, Dominique Dutscher) containing Penicillin-Streptomycin (50 UI/ml and 50 µg/ml) (GIBCO®). HL60 cells were seeded every 2–3 days at 100,000 cells/mL in 5 mL in 25 cm^2^ flasks (BD Falcon). H1975 cells were cultured in RPMI-1640 medium supplemented with 10% Fetal Calf Serum (FCS) (lot n°S11060S181, Dominique Dutscher) and 1% Sodium Pyruvate and Hepes (Lifetechnologies). H1975 cells were seeded twice a week at 300,000 cells/mL in 20 mL in a 75 cm^2^ flask (BD Falcon). All cell lines were incubated at 37 °C and 5% CO_2_.

### Bacteria culture

5 mL of Luberia Broth (ThermoFisher) supplemented with 50 µg/mL of Kanamycin (ThermoFisher) was inoculated with one colony of BL21(DE3) cells transformed with the plasmid pET_eGFP. After overnight growth with agitation at 37 °C, the culture was diluted with fresh medium to reach an absorbancy at 600 nm of about 0.3 and expression was induced by the addition of 0.2 mM Isopropyl β-D-thiogalactoside (IPTG) (ThermoFisher). After 4 hours, cells were diluted to reach the desired dilution. We have assumed that a culture of OD600 of 1.0 equal 5 × 10^8^ cells/mL.

### Plasmid description

A sequence optimized gene for *E. coli* corresponding to eGFP (Genbank gi: 7453572; from MVS. to LYK) followed by a stop codon (TAA) and flanked by suitable restriction sites (5′-*Nde*I and 3′-*Xho*I) was ordered from Sigma/Genewiz. The synthetic gene and pET28a were digested by *Nde*I and *Xho*I and the purified fragments were ligated to create pET_eGFP.

### Microfluidic chip fabrication

Microfluidic devices were prepared from poly (dimethylsiloxane) (PDMS) by standard soft-lithography techniques^1^. A mold of SU-8 resist (MicroChem) was fabricated on a silicon wafer (NEYCO) by UV exposure (MJB4 contact mask aligner; SUSS MicroTec) through a photolithography mask (Selba) and developed (SU-8 developer; Micro-Chem). Curing agent was added to the PDMS base (Sylgard 184 silicone elastomer kit; DowCorning) to a final concentration of 10% (w/w), mixed, and poured over the mold. Following degassing for several minutes and crosslinking at 75 °C for one hour, the PDMS was peeled off the mold and the input and output ports were punched with a 0.75 mm diameter biopsy punch (WPI). Particles of PDMS were cleared from the ports using Scotch tape, rinsing with Isopropanol and drying with pressurized nitrogen. The structured side of the PDMS slab was bonded to a 75 × 50 × 1.2 mm glass microscope slide (Corning) by exposing both parts to an oxygen plasma (PICO, Diener) and pressing them together. Finally, an additional hydrophobic surface coating was applied to the microfluidic channel walls by injecting the completed device with Aquapel glass treatment (PPG Industries) and then purging the liquid with nitrogen gas. For the electro-microfluidic chips the PDMS device is plasma bonded to the non-conductive side of a 75 × 50 × 1.1 mm Indium Tin Oxide glass (ITO, Delta Technologies). The conductive side of the ITO glass is used as a counter electrode. Electrodes are incorporated into the system by filing the micro channels with a low-melting point solder (Indalloy 19, Indium corporation).

## Electronic supplementary material

Cell encapsulation

Reagants injection

Supplementary Information
