# Author Correction: High throughput single cell counting in droplet-based microfluidics

**DOI:** 10.1038/s41598-020-66763-7

**Published:** 2020-06-17

**Authors:** Heng Lu, Ouriel Caen, Jeremy Vrignon, Eleonora Zonta, Zakaria El Harrak, Philippe Nizard, Jean-Christophe Baret, Valérie Taly

**Affiliations:** 1 INSERM UMR-S1147, CNRS SNC5014, Paris Descartes University, Equipe labellisée Ligue Nationale contre le cancer, Paris, France; 2 CNRS, Univ. Bordeaux, CRPP, UPR 8641, 115 Avenue Schweitzer, 33600 Pessac, France

Correction to: *Scientific Reports* 10.1038/s41598-017-01454-4, published online 02 May 2017

In the original version of this Article, there were errors in Affiliation 1 which was incorrectly listed as ‘Université Paris Sorbonne Cité, Inserm UMR-S1147, 45 rue des Saints-Pères, 75270, Paris, France’. The correct affiliation is listed below:

INSERM UMR-S1147, CNRS SNC5014, Paris Descartes University, Equipe labellisée Ligue Nationale contre le cancer, Paris, France

Furthermore the HTML version of this Article contained an error in the order of the two supplementary videos.

These errors have now been corrected in the PDF and HTML versions of the Article, and in the accompanying Supplementary Information files.

