# Supplementary material for: High throughput single cell counting in droplet-based microfluidics
Source: Sci Rep. 2017 May 2;7:1366. doi: 10.1038/s41598-017-01454-4 (PMC5431057; doi:10.1038/s41598-017-01454-4)
Supplement: Supplementary file 3 — Supplementary Information [file 41598_2017_1454_MOESM3_ESM.pdf]

# High throughput single cell counting in droplet-based microfluidics

**Heng Lu[1,†], Ouriel Caen[1,†], Jeremy Vrignon[2], Eleonora Zonta[1], Zakaria El Harrak[1], Philippe Nizard[1], Jean-Christophe Baret[2,*] and Valérie Taly[1,**]**

[1] INSERM UMR-S1147, CNRS SNC5014, Paris Descartes University, Equipe labellisée Ligue Nationale contre le cancer, Paris, France

[2] CNRS, Univ. Bordeaux, CRPP3, UPR 8641, 115 Avenue Schweitzer, 33600 Pessac, France

[*] jean-christophe.baret@u-bordeaux.fr

[**] valerie.taly@parisdescartes.fr

[+] these authors contributed equally to this work

# Supplementary Information

## Supplementary Table S1

| Characterization of cell occupancy | | Results | | | References |
|---|---|---|---|---|---|
| Average cell per droplet ($\lambda$) | Counting method | Number of cells | Applications | Number of droplets analyzed | |
| 0.1 | High speed camera | NI | RNA-seq | 30,000 | [1] |
| 0.1 | High speed camera | NI | RNA-seq | 4,480,800 | [2] |
| 0.1 | NI | NI | ChIP-seq | 96,000 | [3] |
| 0.75 | Fluorescence imaging | NI | Cell sorting | 824 | [4] |
| 0.19; 0.54 | NI | NI | High throughput screening | >50,000 | [5] |
| 0.3 | NI | NI | High throughput screening | >1,000,000 | [6] |
| 0.01-0.05 | NI | NI | RT-PCR, Taqman | 47,078 | [7] |
| 0.3-0.5 | NI | NI | Gene reporter assay | 60,000 | [8] |
| 1 | LIF | 1000 | Cell viability assay | 10,000 | [9] |
| 0.1-0.5 | High speed camera | 350 | Cell viability assay | NI | [10] |
| 0.31-2.5 | High speed camera | 120 | Cell viability assay | NI | [11] |

**Table S1.** Recent single-cell analysis studies using droplet-based microfluidics. The number of cells which were counted for the characterization of cell occupancy is highlighted in red. NI, not indicated. LIF, laser induced fluorescence.

## Supplementary Figure S1

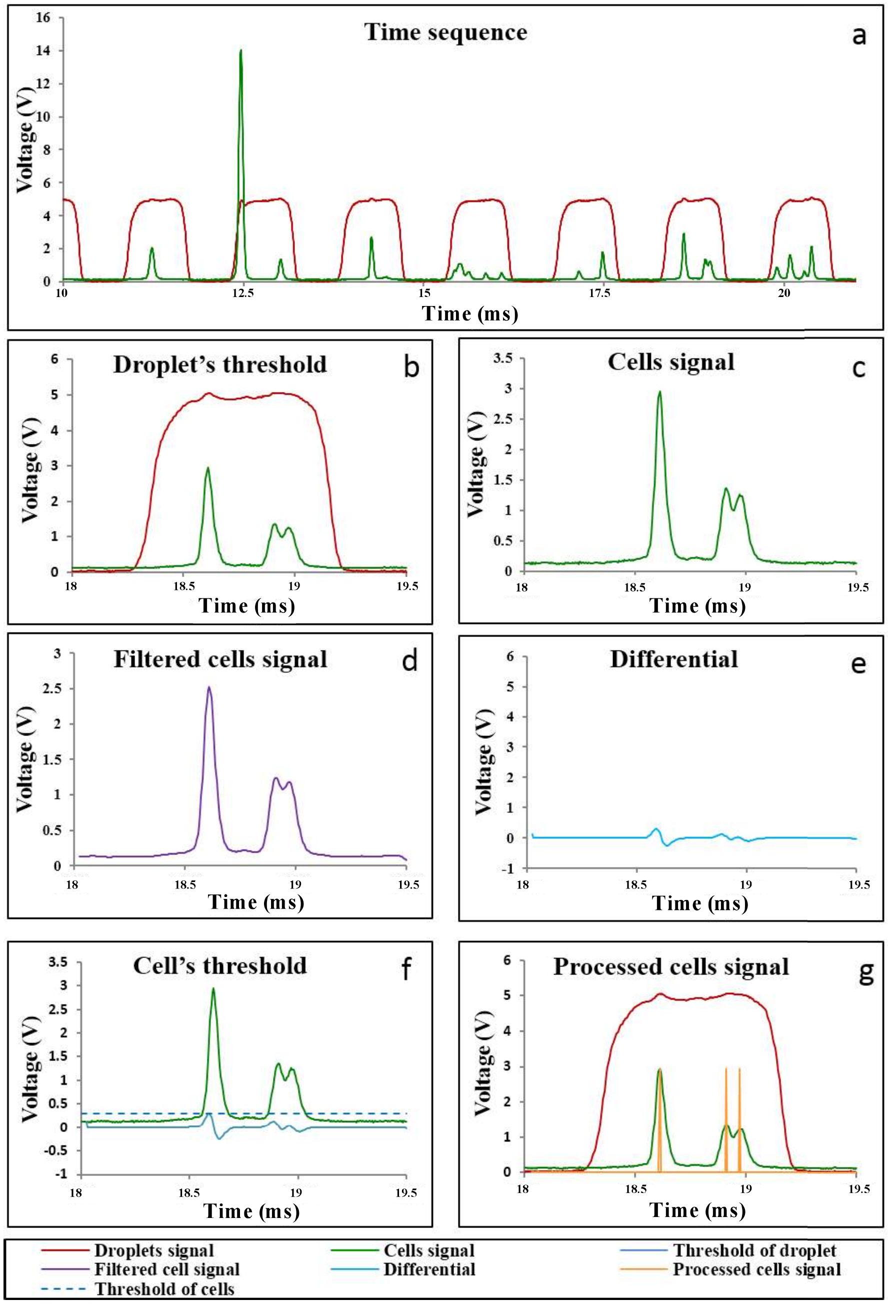


**Figure S1.** Illustration of signal processing. (a) Fluorescent signals of droplets and cells to be analyzed by the cell counting process. This process is divided in 6 parts: the droplet domain is defined by applying a droplet threshold (b); within each droplet defined in (b), the cell signal is isolated (c) and filtered by convoluting a triangle window to withdraw noise (d). A first order differential of the filtered signal is applied to identify the local maximal values (e); then by combining local maximal values identification and cell threshold as a criteria to define signal peaks (f). The number of cells per droplet is then enumerated as signal peaks within the interval of each droplet (g).

**Supplementary Figure S2**

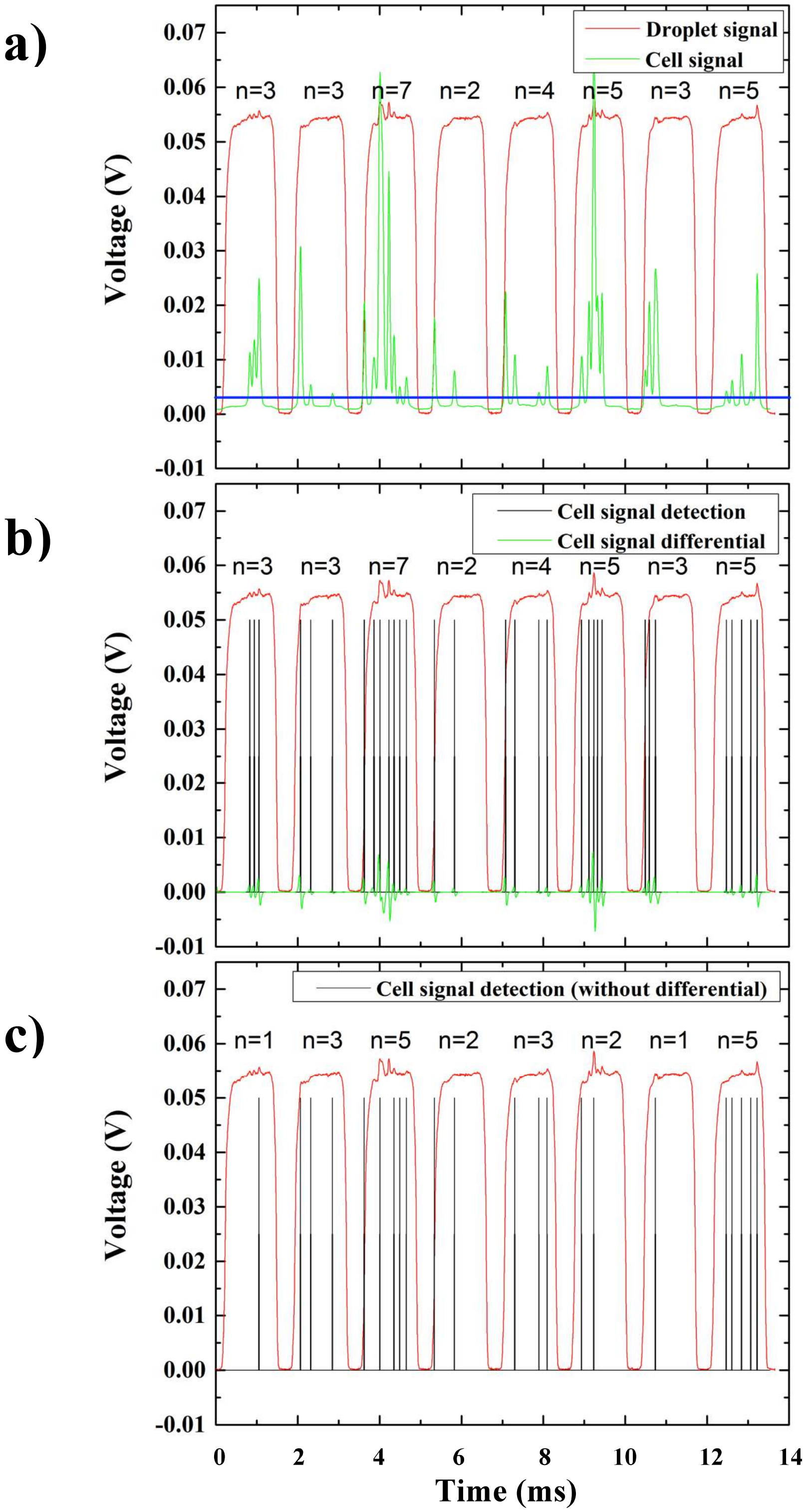


**Figure S2.** Time sequence analysis with and without differential-based cell signal detection. a) Original fluorescence signals. Blue line: cell threshold defined for signal analysis. n: count of cells per droplet. b) Cell signal detection resulting from the differential-based procedure. Algorithm-extracted cell count per droplet is consistent with the expected count (a)). c) Cell signal detection without the differential-based procedure. A discrepancy is observed between extracted and expected cell counts.

**Supplementary Figure S3**

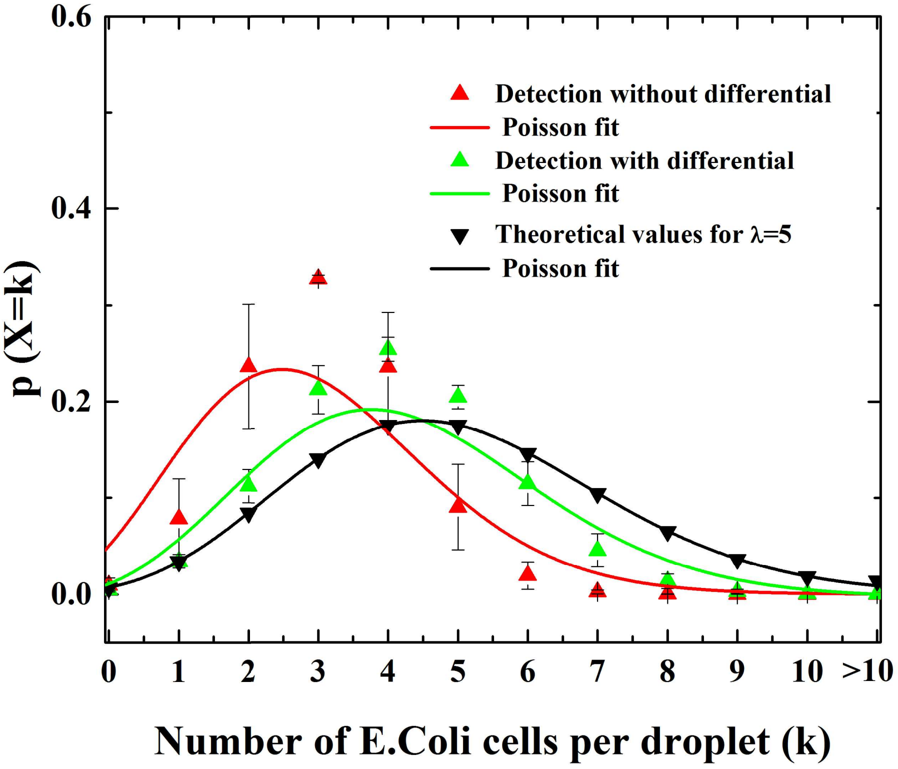


**Figure S3.** Poisson distribution analysis with and without differential-based cell signal detection. Cell density was adjusted to $4.2x10^8$ cells/mL such that $\lambda_{theo}$=5 is expected. mean±s.d for n=2. Cell signal detection resulting from the differential-based procedure resulted in $\lambda_{exp}$=4.08 (average of 731,518 cells counted out of 179,058 droplets). Cell distribution fit : $\lambda_{fit}$=4.2 with $R^2$=0.9. Cell signal detection without the differential-based procedure resulted in $\lambda_{exp}$=3.06 (average of 548,128 cells counted out of 179,058 droplets). Cell distribution fit : $\lambda_{fit}$=3.2 with $R^2$=0.84.

# Supplementary Figure S4

```
clear all;
close all;
clc;

M=wavread('lambda01_0_16b');              %% Loading of the WAV file containing droplets' and cells' signal
green=M(:,1);
red=M(:,2);
drop_s=red;
cell_s=green;
f=1:10000;
plot(f,drop_s(f),'r',f,cell_s(f),'g');    %% Displaying a time sequence graph of droplets' and cells' signal

%%%%%%%%%%%%%%%%%%%%%%%%%%%%%%%%%%%%%%%%%%%%%%%%%%%%%%%%%%%%%%%%%%%%%%%%%%%%%%%%%%%%%%%%%%%%%%%%%%%%%%%%%%%%%%%%%%%%%%%%%%%%

droplet_threshold=0.002;                  %% Defining the threshold of droplets' signal
droplet_number=0;
drop_width=0;
drop_min_width=100;                       %% Defining the minimal droplets' width
%%%%%%%%%%%%%%%%%%%%%%%%%%%%%%%%%%%%%%%%%%%%%%%%%%%%%%%%%%%%%%%%%%%%%%%%%%%%%
for i=1:length(drop_s)                                                     %%
    if i+1<length(drop_s)                                                  %%
        if drop_s(i)>droplet_threshold                                     %%
            drop_width=drop_width+1;                                       %%
            if drop_width>drop_min_width && drop_s(i+1)<droplet_threshold  %%
                droplet_number=droplet_number+1;                           %% Counting the number of droplets
            end                                                            %%
        else                                                               %%
            drop_width=0;                                                  %%
        end                                                                %%
    end                                                                    %%
end                                                                        %%
%%%%%%%%%%%%%%%%%%%%%%%%%%%%%%%%%%%%%%%%%%%%%%%%%%%%%%%%%%%%%%%%%%%%%%%%%%%%%
drop_width=0;
%%%%%%%%%%%%%%%%%%%%%%%%%%%%%%%%%%%%%%%%%%%%%%%%%%%%%%%%%%%%%%%%%%%%%%%%%%%%%%%%%%%%%%%%%%%%%%%%%%%%%%%%%%%%%%%%%%%%%%%%%%%
Cell_index=cell(droplet_number,1);                                                                                     %%
drop_width_mat=zeros(droplet_number,1);                                                                                %%
droplet_index=0;                                                                                                       %%
for i=1:length(drop_s)                                                                                                 %%
    if i+1<length(drop_s) && droplet_index<droplet_number && i-drop_min_width-1>0 && i+drop_min_width+1<length(drop_s)%%
        if drop_s(i)>droplet_threshold                                                                                 %%
            drop_width=drop_width+1;                                                                                   %%
            if drop_width>drop_min_width                                                                               %%
                if drop_s(i-drop_min_width-1)<droplet_threshold                                                        %%
                    droplet_index=droplet_index+1;                                                                     %%
                end                                                                                                    %%
                if droplet_index>0                                                                                     %%
                    Cell_index{droplet_index}=[Cell_index{droplet_index} i];                                           %% Mapping the droplet-passing time
                    if drop_s(i+1)<droplet_threshold                                                                   %% points
                        drop_width_mat(droplet_index)=drop_width;                                                      %%
                    end                                                                                                %%
                end                                                                                                    %%
            end                                                                                                        %%
        else                                                                                                           %%
            drop_width=0;                                                                                              %%
        end                                                                                                            %%
    end                                                                                                                %%
end                                                                                                                    %%
%%%%%%%%%%%%%%%%%%%%%%%%%%%%%%%%%%%%%%%%%%%%%%%%%%%%%%%%%%%%%%%%%%%%%%%%%%%%%%%%%%%%%%%%%%%%%%%%%%%%%%%%%%%%%%%%%%%%%%%%%%%

if isempty(Cell_index{droplet_number})
    Cell_index=Cell_index(1:end-1);
    droplet_number=droplet_number-1;                                           %% If the last droplet signal is incomplete, it will be deleted
end

%%%%%%%%%%%%%%%%%%%%%%%%%%%%%%%%%%%%%%%%%%%%%%%%%%%%%%%%%%%%%%%%%%%%%%%%%%%%%%%%%%%%%%%%%
for i=1:droplet_number                                                                 %%
    Cell_index{i}=[Cell_index{i}(1)-drop_min_width:Cell_index{i}(1) Cell_index{i}]; %% Concatenating the droplet-passing time points
end                                                                                    %% with minimal droplet width points
%%%%%%%%%%%%%%%%%%%%%%%%%%%%%%%%%%%%%%%%%%%%%%%%%%%%%%%%%%%%%%%%%%%%%%%%%%%%%%%%%%%%%%%%%

%%%%%%%%%%%%%%%%%%%%%%%%%%%%%%%%%%%%%%%%%%%%%%%%%%%%%%%%%%%%%%%%%%%%%%%%%%%%%%%%%%%%%%%%%%%%%%%%%%%%%%%%%%%%%%%%%
                                                                                                              %%
cell_threshold=0.002;                                                                                         %% Defining the the threshold of cell signal
kernel=[1:100 sort(1:99,'descend')];                                                                          %% Defining the triangular convolution filter
cell_filter=cell(size(Cell_index));                                                                           %%
cell_filter_diff=cell(size(Cell_index));                                                                      %%
N_cell=zeros(droplet_number,1);                                                                               %%
for i=1:droplet_number                                                                                        %%
     cell_filter{i}=conv(cell_s(Cell_index{i}),kernel,'same')/sum(kernel);                                    %% Convoluting of cell signal with a triangular filter
     cell_filter_diff{i}=diff(cell_filter{i});                                                                %% First-order derivative of filtered cell signal
     cell_filter_diff{i}=[cell_filter_diff{i}(1);cell_filter_diff{i}];                                        %%
     for j=1:length(Cell_index{i})-1                                                                          %%
         if cell_filter_diff{i}(j)>0 && cell_filter_diff{i}(j+1)<0 && cell_s(Cell_index{i}(j))>cell_threshold %% Cell pic criteria: precedent
                 N_cell(i)=N_cell(i)+1;                                                                       %% derivative point is positive,
         end                                                                                                  %% successive point is negative
     end                                                                                                      %% and cell signal is over the
 end                                                                                                          %% cell signal threshold
                                                                                                              %%
                                                                                                              %%
                                                                                                              %%
 %%%%%%%%%%%%%%%%%%%%%%%%%%%%%%%%%%%%%%%%%%%%%%%%%%%%%%%%%%%%%%%%%%%%%%%%%%%%%%%%%%%%%%%%%%%%%%%%%%%%%%%%%%%%%%%%
                                                                                                              %%
 Cell_distribution=zeros(3,6);                                                                                %% Extracting cell distribution in droplets
 Cell_distribution(1,:)=0:5;                                                                                  %%
 for i=1:length(N_cell)                                                                                       %%
     switch N_cell(i)                                                                                         %%
         case 0                                                                                               %%
             Cell_distribution(2,1)=Cell_distribution(2,1)+1;                                                 %%
         case 1                                                                                               %%
             Cell_distribution(2,2)=Cell_distribution(2,2)+1;                                                 %%
         case 2                                                                                               %%
             Cell_distribution(2,3)=Cell_distribution(2,3)+1;                                                 %%
         case 3                                                                                               %%
             Cell_distribution(2,4)=Cell_distribution(2,4)+1;                                                 %%
         case 4                                                                                               %%
             Cell_distribution(2,5)=Cell_distribution(2,5)+1;                                                 %%
         otherwise                                                                                            %%
             Cell_distribution(2,6)=Cell_distribution(2,6)+1;                                                 %%
     end                                                                                                      %%
 end                                                                                                          %%
 Cell_distribution(3,:)=Cell_distribution(2,:)/sum(Cell_distribution(2,:));                                   %%
 bar(Cell_distribution(1,:),Cell_distribution(3,:));                                                          %%
 Cell_distribution=transpose(Cell_distribution);                                                              %%
                                                                                                              %%
 %%%%%%%%%%%%%%%%%%%%%%%%%%%%%%%%%%%%%%%%%%%%%%%%%%%%%%%%%%%%%%%%%%%%%%%%%%%%%%%%%%%%%%%%%%%%%%%%%%%%%%%%%%%%%%%%
```

**Figure S4.** MATLAB script used for signal processing. Code comments are preceded by “%%”.

## Supplementary Figure S5

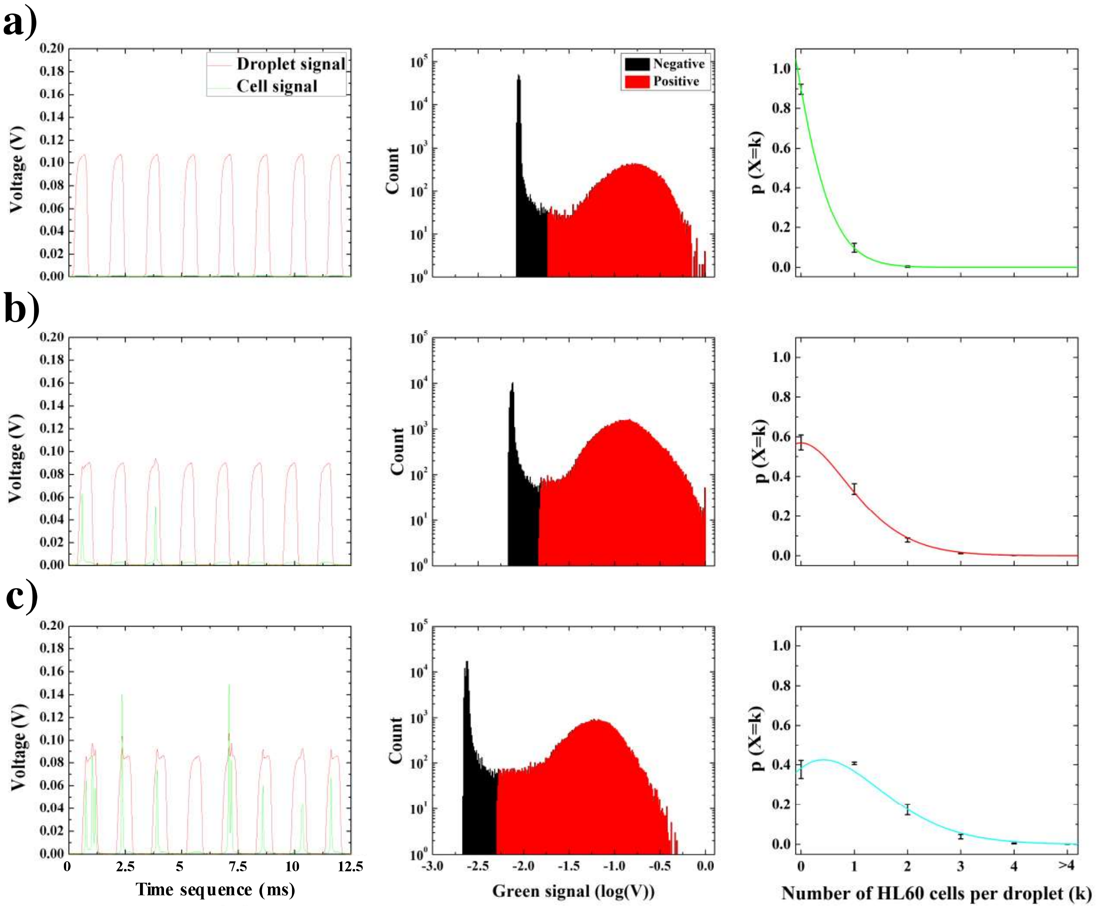


**Figure S5.** Counting of HL60 cells. (a)-(c) From left to right: time sequences of red and green fluorescence signals, histograms of the green fluorescence signal depicting negative and positive cell count events, and cell distribution in droplets (mean±s.d for n=3; Poisson fit is plotted as a straight line). (a) Cell density was adjusted to $2x10^5$ cells/mL such that expected theoretical cell to droplet ratio ($\lambda_{theo}$) is $\lambda_{theo}$=0.1 (given that droplet's volume is 500 pL). In average 18,355±5,112 cells were counted out of 173,403±47,696 droplets resulting an experimental cell to droplet ratio ($\lambda_{exp}$) $\lambda_{exp}$=0.1±0.03. Cell distribution fitted $\lambda_{fit}$=0.1±($7.4x10^{-4}$) with $R^2$=0.99. (b) Cell density was adjusted to $10^6$ cells/mL such that $\lambda_{theo}$=0.5 is expected. In average 83,916±7,888 cells were counted out of 157,478±38,753 droplets resulting in $\lambda_{exp}$=0.53±0.05. Cell distribution fitted $\lambda_{fit}$=0.56±0.01 with $R^2$=0.99. (c) Cell density was adjusted to $2x10^6$ cells/mL such that $\lambda_{theo}$=1 is expected. In average 167,248±19,111 cells were counted out of 188,499±63,428 droplets resulting in $\lambda_{exp}$=0.89±0.1. Cell distribution fitted $\lambda_{fit}$=0.96±0.01 with $R^2$=0.98.

## Supplementary Figure S6

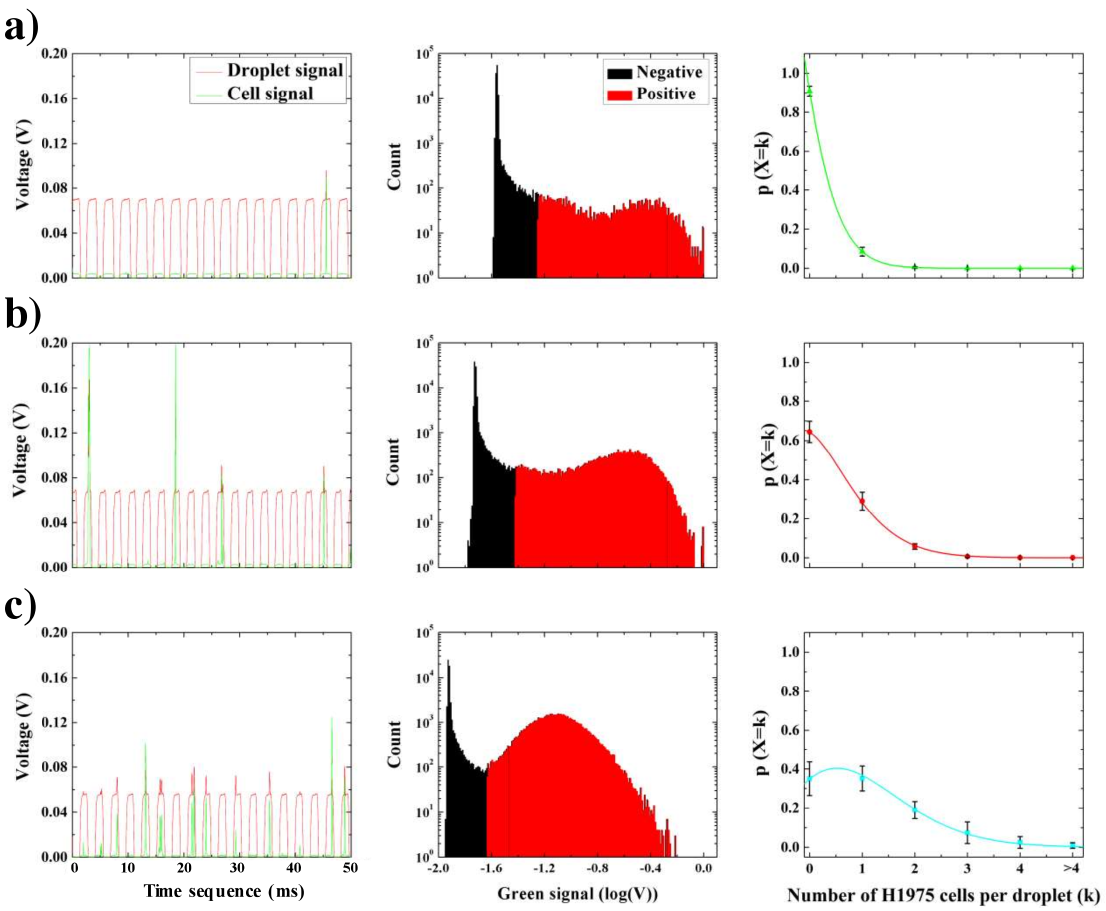


**Figure S6.** Counting of H1975 cells. (a)-(c) From left to right: time sequences of red and green fluorescence signals, histograms of the green fluorescence signal depicting negative and positive cell count events, and cell distribution in droplets (mean±s.d for n=3; Poisson fit is plotted as a straight line). (a) Cell density was adjusted to 2x$10^5$ cells/mL such that expected theoretical cell to droplet ratio ($\lambda_{theo}$) is $\lambda_{theo}$=0.1 (given that droplet's volume is 500 pL). In average 9,779±2,925 cells were counted out of 101,203±11,583 droplets resulting an experimental cell to droplet ratio ($\lambda_{exp}$) $\lambda_{exp}$=0.1±0.03. Cell distribution fitted $\lambda_{fit}$=0.1±0.006 with $R^2$=0.99. (b) Cell density was adjusted to $10^6$ cells/mL such that $\lambda_{theo}$=0.5 is expected. In average 48,513±10,036 cells were counted out of 113,308±3,720 droplets resulting in $\lambda_{exp}$=0.43±0.09. Cell distribution fitted $\lambda_{fit}$=0.44±0.006 with $R^2$=0.99. (c) Cell density was adjusted to 2x$10^6$ cells/mL such that $\lambda_{theo}$=1 is expected. In average 120,849±50,055 cells were counted out of 114,431±3361 droplets resulting in $\lambda_{exp}$=1.06±0.44. Cell distribution fitted $\lambda_{fit}$=1±0.02 with $R^2$=0.99.

**Supplementary Figure S7**

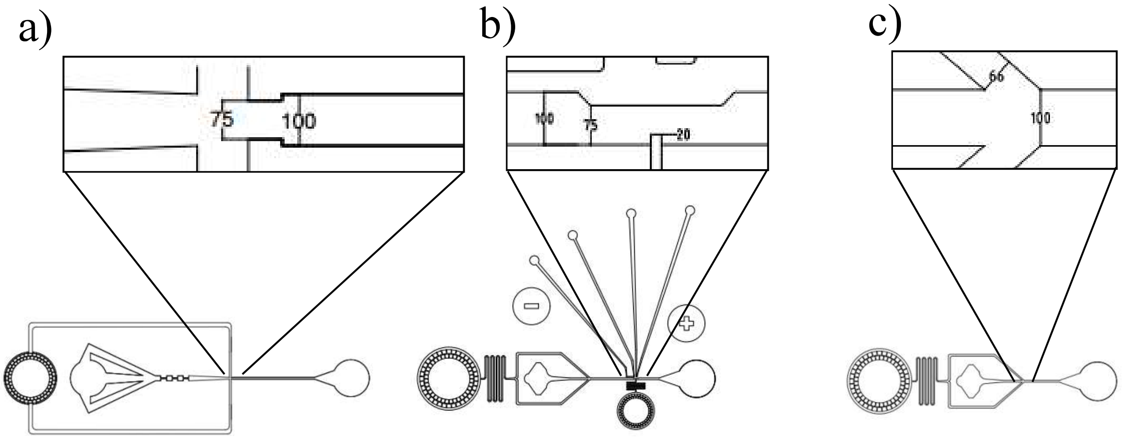


**Figure S7.** Microfluidic chips designs. a) Mammalian cells' encapsulator (channel's depth=70 μm); b) Bacteria cells' encapsulator (channel's depth=25 μm); c) Picoinjector for cell staining (channel's depth=70 μm).

**Supplementary Figure S8**

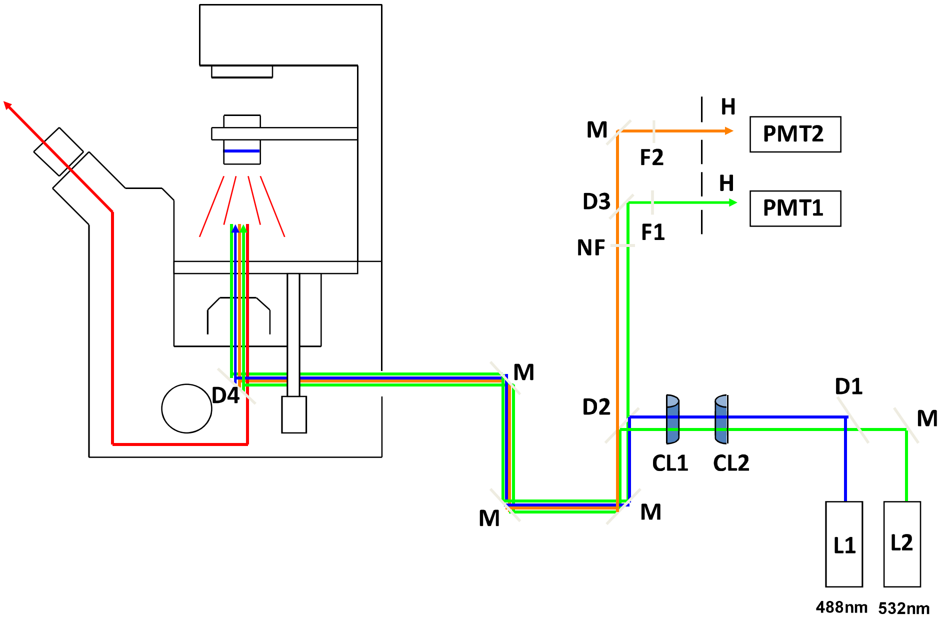


**Figure S8.** Optical set-up. Two lasers (L1, L2) focused in the microfluidic channels allow the excitation of droplets and cells fluorophores. Emission is measured simultaneously on two photomultiplier tubes (PMT) in the green and orange windows of the light spectrum. Two cylindrical lenses (CL1, CL2) allow to transform the laser spot in laser line configuration enabling the excitation of cells independently of their position in the droplets. L1, L2: lasers (Cobolt 06-MLD 488nm, Cobolt 06-DPL 532nm; Cobolt). CL1, CL2: cylindrical lenses (CL1: LJ1878L2-A, CL2: LJ1653L1-A; Thorlabs). D1, D2, D3, D4: dichroic bimsplitters (D1: Di01-R488-25x36, D2: Di01-R405/488/532/635-25x36x5.0, D3:FF562-Di03-25x36, D4: FF605-Di02; Semrock). F1, F2: bandpass filters (F1: FF01-524/24-25, F2: FF01-575/25-25; Semrock). NF: notch filter (NF01-405/488/532/635 25x5.0, Semrock). M: mirrors (BB1-E02, Thorlabs). H: diaphragm (SM1D12D).

## Supplementary Figure S9

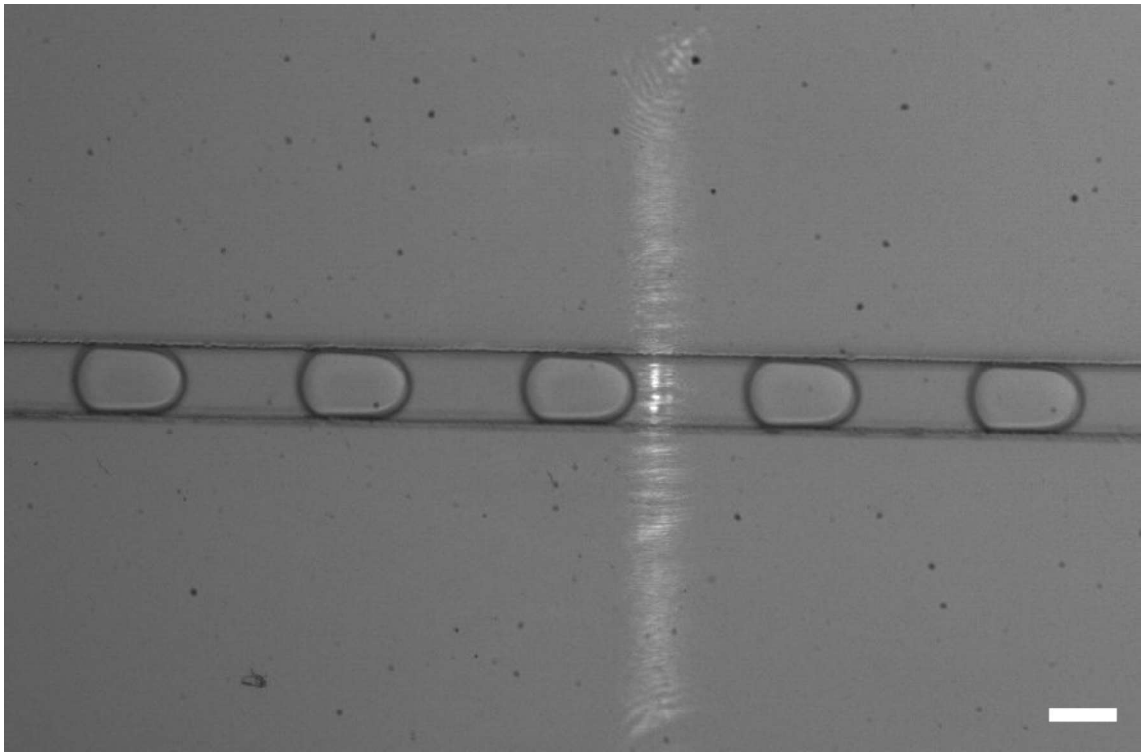

**Figure S9.** Bright field image of the laser line excitation area. A full droplet fluorescence scan is performed as laser line is larger than the channel width. Scale ber: 100µm.

## Supplementary Video S1

**Video S1.** Encapsulation of human H1975 cells at $2x10^6$ cells/mL density in ~500 pL droplets. Video is slowed down 170 times.

## Supplementary Video S2

**Video S2.** Injection of the viability assay in droplets. Video is slowed down 114 times.

**Reference**


1. Klein, Allon M., et al., *Droplet Barcoding for Single-Cell Transcriptomics Applied to Embryonic Stem Cells.* Cell. **161**(5): p. 1187-1201.

2. Macosko, Evan Z., et al., *Highly Parallel Genome-wide Expression Profiling of Individual Cells Using Nanoliter Droplets.* Cell, 2015. **161**(5): p. 1202-1214.

3. Rotem, A., et al., *Single-cell ChIP-seq reveals cell subpopulations defined by chromatin state.* Nat Biotech, 2015. **33**(11): p. 1165-1172.

4. Hu, H., D. Eustace, and C.A. Merten, *Efficient cell pairing in droplets using dual-color sorting.* Lab on a Chip, 2015. **15**(20): p. 3989-3993.

5. Wang, B.L., et al., *Microfluidic high-throughput culturing of single cells for selection based on extracellular metabolite production or consumption.* Nat Biotechnol, 2014. **32**(5): p. 473-8.

6. Mazutis, L., et al., *Single-cell analysis and sorting using droplet-based microfluidics.* Nat Protoc, 2013. **8**(5): p. 870-91.

7. Eastburn, D.J., A. Sciambi, and A.R. Abate, *Ultrahigh-throughput Mammalian single-cell reverse-transcriptase polymerase chain reaction in microfluidic drops.* Anal Chem, 2013. **85**(16): p. 8016-21.

8. Baret, J.C., et al., *Quantitative cell-based reporter gene assays using droplet-based microfluidics.* Chem Biol, 2010. **17**(5): p. 528-36.

9. Brouzes, E., et al., *Droplet microfluidic technology for single-cell high-throughput screening.* Proc Natl Acad Sci U S A, 2009. **106**(34): p. 14195-200.

10. Koster, S., et al., *Drop-based microfluidic devices for encapsulation of single cells.* Lab on a Chip, 2008. **8**(7): p. 1110-5.

11. Clausell-Tormos, J., et al., *Droplet-based microfluidic platforms for the encapsulation and screening of Mammalian cells and multicellular organisms.* Chem Biol, 2008. **15**(5): p. 427-37.
